# Novel NIBS in psychiatry: Unveiling TUS and TI for research and treatment

**DOI:** 10.1177/23982128251322241

**Published:** 2025-03-14

**Authors:** Faissal Sharif, Catherine J Harmer, Miriam C. Klein-Flügge, Huiling Tan

**Affiliations:** 1 MRC Brain Network Dynamics Unit, Nuffield Department of Clinical Neurosciences, University of Oxford, Oxford, UK; 2 Department of Psychiatry, University of Oxford, Warneford Hospital, Oxford, UK; 3 Department of Experimental Psychology, University of Oxford, Oxford, UK; 4 Wellcome Centre for Integrative Neuroimaging (WIN), Centre for Functional MRI of the Brain (FMRIB), Nuffield Department of Clinical Neurosciences, John Radcliffe Hospital, University of Oxford, Oxford, UK

**Keywords:** Non-invasive brain stimulation, neuromodulation, neurotechnology, neurostimulation, temporal interference stimulation, transcranial focused ultrasound stimulation, psychiatry, mental disorders, transcranial ultrasonic stimulation, depression

## Abstract

Mental disorders pose a significant global burden and constitute a major cause of disability worldwide. Despite strides in treatment, a substantial number of patients do not respond adequately, underscoring the urgency for innovative approaches. Traditional non-invasive brain stimulation techniques show promise, yet grapple with challenges regarding efficacy and specificity. Variations in mechanistic understanding and reliability among non-invasive brain stimulation methods are common, with limited spatial precision and physical constraints hindering the ability to target subcortical areas often implicated in the disease aetiology. Novel techniques such as transcranial ultrasonic stimulation and temporal interference stimulation have gained notable momentum in recent years, possibly addressing these shortcomings. Transcranial ultrasonic stimulation (TUS) offers exceptional spatial precision and deeper penetration compared with conventional electrical and magnetic stimulation techniques. Studies targeting a diverse array of brain regions have shown its potential to affect neuronal excitability, functional connectivity and symptoms of psychiatric disorders such as major depressive disorder. Nevertheless, challenges such as target planning and addressing acoustic interactions with the skull must be tackled for its widespread adoption in research and potentially clinical settings. Similar to transcranial ultrasonic stimulation, temporal interference (TI) stimulation offers the potential to target deeper subcortical areas compared with traditional non-invasive brain stimulation, albeit requiring a comparatively higher current for equivalent neural effects. Promising yet still sparse research highlights TI’s potential to selectively modulate neuronal activity, showing potential for its utility in psychiatry. Overall, recent strides in non-invasive brain stimulation methods like transcranial ultrasonic stimulation and temporal interference stimulation not only open new research avenues but also hold potential as effective treatments in psychiatry. However, realising their full potential necessitates addressing practical challenges and optimising their application effectively.

## Introduction

Mental disorders significantly contribute to disability and disease burden globally (Friedrich, 2017; Wittchen et al., 2011). The World Health Organization reports that over 25% of individuals will encounter a mental health disorder during their lifetime. Presently, approximately 970 million people are living with a mental disorder, equating to 1 in 8 individuals (Mental Disorders, 2023). Despite the advances in psychiatry in the past decades, a large number of affected individuals still do not respond to current treatments or struggle to achieve full remission.

Psychiatric disorders such as major depressive disorder (MDD) are associated with structural and functional changes in neural circuits relevant to emotion and cognition (Marx et al., 2023). These circuits can be modulated using antidepressants and other medications but may be more specifically targeted using brain stimulation methods. Among these treatments are brain stimulation methods with a long-standing history in psychiatry, notably electroconvulsive therapy (ECT), which has been in use for over 80 years, mostly for schizophrenia and severe MDD (National Institute for Health and Care Excellence (NICE), 2009). ECT is a medical procedure in which an electric current is passed through the brain via electrodes placed on the temples, inducing a controlled seizure lasting for about 1 min (Gazdag and Ungvari, 2019). In the past two decades, several other brain stimulation methods were approved for use in psychiatric disorders. Vagus nerve stimulation (VNS) is a method in which an implanted pulse generator delivers electrical impulses to the left vagus nerve (Kamel et al., 2022). In 2005, VNS was approved by the Food and Drug Administration (FDA) for severe, recurrent unipolar and bipolar depression with mixed results regarding its efficacy (Kamel et al., 2022; O’Reardon et al., 2006; Vlaicu and Bustuchina Vlaicu, 2020). A more recent, non-invasive form known as transcutaneous VNS (tVNS) is currently being investigated for use in MDD and post-traumatic stress disorder (PTSD), among other disorders (Yap et al., 2020). In 2008, the FDA-approved repetitive transcranial magnetic stimulation (rTMS) to treat MDD, which was extended to obsessive-compulsive disorder (OCD) in 2018 (Office of the Commissioner, 2020). Beyond the United States, rTMS targeting the dorsolateral prefrontal cortex (dlPFC) has gained regulatory approval for depression treatment in several countries, including Canada, Australia and Germany, reflecting its global recognition as a viable therapy for treatment-resistant depression (Bourla et al., 2020; McClintock et al., 2018).

rTMS is a non-invasive technique that employs repeated low-intensity magnetic pulses to targeted brain areas. It has demonstrated efficacy in treating MDD with numerous studies confirming its therapeutic benefits (McClintock et al., 2018; Rachid, 2018; Voigt et al., 2019). Notably, a large-scale randomised controlled trial (RCT) demonstrated sustained efficacy of rTMS targeting the dlPFC, with significant improvements in depressive symptoms persisting over a 26-week period (Morriss et al., 2024). This reinforces the importance of precise targeting of the dlPFC, as its stimulation is believed to exert its effects indirectly by modulating deeper structures such as the subgenual anterior cingulate cortex (sgACC) and amygdala, underscoring the advantage of precise techniques that would be able to target deeper areas directly (Fox et al., 2012; Grosshagauer et al., 2024; Ironside et al., 2019; Liston et al., 2014). Recent advancements include accelerated rTMS protocols, which deliver multiple sessions per day, potentially expediting antidepressant responses and improving patient outcomes (Chen et al., 2023; Cole et al., 2022, 2024; Shi et al., 2024). However, individual variability in response persists, and ongoing research aims to optimise stimulation parameters and targeting strategies to enhance long-term effectiveness (Amad and Fovet, 2021).

Transcranial electrical stimulation (tES) including direct current (tDCS), alternating current (tACS) or random current/noise (rtRNS) stimulation allows for non-invasive brain stimulation (NIBS) through the cortex and has been explored in a number of psychiatric disorders, including MDD (Reed and Cohen Kadosh, 2018). While some RCTs report positive effects on neurocognition and depressive symptoms (McClintock et al., 2020; Woodham et al., 2024), other studies have yielded inconsistent results (Aust et al., 2022; Loo et al., 2018; Tao et al., 2024). These discrepancies may stem from variations in stimulation protocols, individual differences in cortical anatomy and methodological challenges (Brunoni et al., 2016). Further research is essential to establish standardised protocols and identify predictors of response to enhance the clinical utility of tES.

As an invasive neuromodulation treatment, deep brain stimulation (DBS) involves implanting an electrode into a predefined, deeper brain area that can then be used for stimulation, and has been traditionally used for movement disorders such as Parkinson’s disease (Davidson et al., 2024). In psychiatry, DBS is most commonly used for Obsessive-compulsory-disorder (OCD), for which it gained FDA clearance in 2009 and is slowly moving into other areas for experimental use, including treatment-resistant depression (TRD) and substance-use disorders (SUDs; Delaloye and Holtzheimer, 2014; Graat et al., 2017; Qu et al., 2019; Widge, 2024). While initial open-label studies showed promising results for TRD, subsequent large multicentre RCTs have yielded mixed outcomes (Dougherty et al., 2015; Holtzheimer et al., 2017; Sobstyl et al., 2022). A comprehensive meta-analysis of 14 open-label studies and three RCTs, involving 233 patients, reported a 56% response rate and a 35% remission rate (Wu et al., 2021). However, over the past two decades, significant advancements have been achieved in target precision in DBS, which have also contributed to refining and supporting the application of other neuromodulation techniques (Lozano and Lipsman, 2013; Meyer et al., 2024; Widge, 2024). Among these, tractography-guided DBS could be a promising avenue to improve targeting (Chan et al., 2024; Gadot et al., 2023). For example, a recent study targeting the subcallosal cingulate cortex (SCC) in 10 patients reported a 90% response rate and a 70% remission rate at 24 weeks, identifying SCC local field potential dynamics as biomarkers for tracking recovery and guiding personalised treatment adjustments (Alagapan et al., 2023). Similarly, an RCT targeting the bed nucleus of the stria terminalis (BNST) and nucleus accumbens (NAcc) demonstrated the efficacy of BNST-NAcc DBS in TRD, with a 50% response rate and a 35% remission rate during the open-label phase and significant improvements in depression, anxiety, quality of life and disability measures during the blinded crossover phase (Voon et al., 2024). Overall, despite recent advancements, DBS in TRD remains a developing therapy and additional work is necessary to refine its effectiveness and establish it as a viable, reliable therapy (Asir et al., 2024; Johnson et al., 2024). The recently launched TRANSCEND clinical trial is a notable multicentre, double-blind, randomised, sham-controlled study evaluating the safety and efficacy of Abbott’s DBS system targeting the SCC, which has received Breakthrough Device designation for TRD by the FDA. Enrolling 100 patients who have failed at least four antidepressant treatments, the trial evaluates its first results 12 months post-surgery (Abbott MediaRoom, n.d.; Clinicaltrials.gov, n.d.).

While DBS has proven effective for certain neurological disorders, its invasive nature and potential side effects underscore the pressing need for the development and refinement of non-invasive alternatives with potentially fewer side effects. NIBS can minimise risks and expand accessibility, paving the way for safer and more widely applicable interventions in the field of neuromodulation.

**Figure 1.**
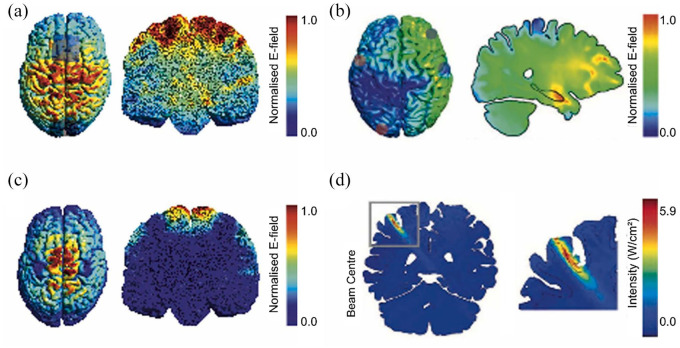
Spatial specificity in non-invasive brain stimulation methods based on simulations. (a) Simulated patterns of spatial specificity for transcranial electrical stimulation, (b) temporal interference stimulation (TI), (c) transcranial magnetic stimulation (tES) and (d) transcranial ultrasonic stimulation (TUS). Source: Nasr et al. (2022).

Despite their numerous benefits over invasive methods, NIBS methods display huge disparities in their utility for both clinical and research settings. This major challenge has been highlighted by a review by Nasr et al. (2022) who compared established and emerging NIBS methods based on their *spatial specificity*, *mechanical specificity* and *robustness. Spatial specificity* pertains to the degree to which the impact of stimulation on neural activity is confined to the intended target brain region (Figure 1). *Mechanical specificity* describes the extent to which the influence of stimulation on neural activity can be attributed to a specific cellular or molecular mechanism. *Robustness* refers to the reliability and replicability of the effects of stimulation on neural activity, as well as its impact on functional and behavioural outcomes (Nasr et al., 2022). Most electrical stimulation methods have the disadvantage of currents being shunted through the scalp and diffusing before reaching the intended target, limiting their *spatial specificity* (Vöröslakos et al., 2018). Therefore, the ability of established NIBS methods to reach subcortical areas often implicated in psychiatric disorders is vastly limited.

It is further challenging to infer mechanistic causality from these stimulation methods alone, as there are many confounding variables between the generation of an electric field, the evoking of neural activity, activation of connected nodes of the network and behavioural outcomes. Controlling for all these factors is not always possible, often due to technical constraints, limiting the ability of NIBS studies to establish clear cause-effect relationships and thus exhibiting high *mechanistic specificity* (Bergmann and Hartwigsen, 2021). Furthermore, *robustness* remains a challenge due to high inter- and intraindividual variability paired with small effect sizes and common replicability issues (Bergmann and Hartwigsen, 2021; Nasr et al., 2022).

Concurrently, there is a heightened interest in novel NIBS methods promising superior spatial and mechanistic specificity such as transcranial ultrasonic stimulation (TUS) and temporal interference stimulation (TI). These methods not only open up avenues for further research but also hold significant potential as viable treatment modalities for a spectrum of psychiatric conditions.

## Transcranial Ultrasonic Stimulation (TUS)

Initially focused on tissue ablation, there is now a surge in neuromodulation applications of TUS which hold the potential for higher spatial specificity and deeper penetration than any other NIBS. Specifically, TUS has been shown to have a spatial resolution of a few millimetres at variable depths (Bystritsky and Korb, 2015; Legon et al., 2020; Nasr et al., 2022; Rabut et al., 2020). Therefore, TUS exhibits the highest spatial specificity among NIBS paradigms, surpassing both electrical and magnetic stimulation methods. Concerning its robustness, several reviews of human TUS studies indicate its potential while also highlighting inherent variability in outcomes arising from individual differences in skull anatomy, acoustic properties and the precision of targeting (Bault et al., 2024; Lee et al., 2024; Pellow et al., 2024; Sarica et al., 2022). Recent proposals for improving the standards and replicability of TUS findings will likely help this relatively young field yield more robust effects across individuals and studies (Klein-Flügge et al., 2024; Martin et al., 2024a; Murphy et al., 2025). Studies have largely operated within the safety standards set by the International Consortium for Transcranial Ultrasonic Stimulation Safety and Standards (ITRUSST) and thus far been well tolerated in hundreds of sessions that were carried out over the past few years by different research groups (Legon et al., 2020; Martin et al., 2024a; Pasquinelli et al., 2019). However, potential risks include acoustic cavitation, particularly if safety limits for mechanical index are exceeded (Aubry et al., 2023). Importantly, individuals with brain calcifications may be at higher risk due to absorption and thus potential thermal changes inside the cranium, particularly if calcifications are present close to the acoustic focus (Lee et al., 2021). ITRUSST has published extensive guidelines to ensure mechanical and thermal safety for each individual (Aubry et al., 2023).

In TUS, a signal generator creates a sinusoidal signal that is then amplified before reaching the transducer, which generates sounds from the oscillating voltage coming from the signal generator. The transducer is placed on the scalp, similarly to how TMS would typically be applied (Bystritsky and Korb, 2015). To achieve the effect of focused ultrasound waves, multiple channels are arranged in a spherical cap configuration which focuses the energy to a central point, typically a few centimetres away from the transducer. Thereby, the extent of concavity in the cap used directly influences the distance to the focal point (Darmani et al., 2022; Di Biase et al., 2019).

Sound is a mechanical or pressure wave that is produced when a given object oscillates at a fundamental frequency. Ultrasound waves start beyond >20 kHz and are not audible to humans. Most TUS experiments employ *acoustic frequency* (*Af*) stimulation between 250 and 700 kHz. The amplitude of the wave affects the peak velocity and displacement of molecular oscillations, while frequency determines their rate. The speed of sound, however, is dictated by the properties of the medium. Intensity is a measure of ultrasound energy in tissue at a given time and is typically set at the range of 3–30 W/cm^2^ intensity of the spatial-peak pulse average (*ISPPA*) (Lee et al., 2021; Rabut et al., 2020). This is well below the safety limit set by the FDA for diagnostic ultrasound devices of 190 W/cm^2^ (Center for Devices and Radiological Health (CDRH), 2023). Typically, TUS is administered in a pulsed mode, employing parameters such as a *pulse length* or *duration* (*PL/PD*) of 1 ms within a *pulse train duration* (*PTD*) or *stimulus duration* (*StimD*) of a few seconds to minutes. The velocity of sound (c) is around 1500 m/s within soft tissue, and it is roughly twice that value in bone. This allows the ultrasound to efficiently reach its target in as little as 40 ms (Darmani et al., 2022; White et al., 2006). The *pulse repetition period* (*PRP*) is determined as the sum of the pulse length and the gap until the next pulse. The *pulse repetition frequency* (*PRF*) is derived by taking the inverse of the *PRP*, establishing the frequency of pulse repetition in TUS per second (Zadeh et al., 2024).

TUS comes with its challenges. One critical consideration in ultrasound applications is minimising the *Target Registration Error (TRE)*, which is the distance between the intended and actual focus. This error is caused by acoustic interactions within the skull which are influenced by its inherent inhomogeneity in its thickness and composition, leading to reflection, refraction and distortion of ultrasound waves (Fitzpatrick and West, 2001; Jung et al., 2019). As sound encounters the skull, a portion undergoes reflection, while the remainder traverses through the skull, potentially with modified direction and phase. Notably, cortical bone (outer layer) exhibits higher absorption, whereas trabecular bone (inner portion) tends to scatter the acoustic waves (Pinton et al., 2012). Cerebrospinal fluid (CSF), white matter and grey matter share similar acoustic properties attributed to their high water content. Ways to mitigate *TRE* will be discussed in later sections. *Porosity (p)* reflects the proportion of void spaces within a material and influences acoustic transmission. In bone, lower porosity corresponds to higher mineral density, leading to greater acoustic impedance and reduced ultrasound transmission (Darmani et al., 2022; Jing et al., 2023). However, higher porosity leads to greater heterogeneity, increasing scattering and energy loss, which reduces transmission at oblique angles (Jing et al., 2023). Furthermore, the frequency of ultrasound waves plays a pivotal role, as higher frequencies lead to increased attenuation by the skull, limiting penetration depth. It is recommended to keep the frequency below 700 kHz to minimise these effects and optimise transmission (White et al., 2006).

On a mechanistic level, TUS most likely works by the *acoustic radiation force (ARF)* of soundwaves, that is, the effects of sound on obstacles. This becomes apparent through several interconnected mechanisms which are still subject to scientific inquiry but have been explored in both in-vitro and in-vivo models (Menz et al., 2019; Tufail et al., 2010; Tyler et al., 2008). One proposed model is the *bilayer sonophore model* which describes the gradual contraction and expansion within the lipid bilayer of neural tissue due to negative pressure-induced cavitation (Plaksin et al., 2014; Ranade et al., 2015; Wahab et al., 2012). The mechanical strain generated by this sets into motion mechanosensitive ion channels (TRPP1/2, TRPC1 and Piezo1) and changes membrane capacitance, exerting a direct influence on neuronal activation and excitability (Blackmore et al., 2023; Darmani et al., 2022; Ranade et al., 2015; Yoo et al., 2022). The ensuing impact on the neural network (e.g. excitatory vs inhibitory effects), however, is contingent upon a multitude of factors. These include the specific stimulation parameters employed, the unique cellular composition of the stimulated region – divergent across various tissue types – and the current state of the overall network (Lord et al., 2024; Murphy et al., 2022; Newman et al., 2024; Plaksin et al., 2014; Yang et al., 2021). These effects are observable through advanced imaging techniques. fMRI illuminates a change in network connectivity, providing insight into the network interactions of acoustic stimulation. On a neurotransmitter level, a decrease in gamma-aminobutyric acid (GABA) in the posterior cingulate but not dorsal anterior cingulate cortex has recently been reported (Yaakub et al., 2023). The same study suggested the reduced GABA content as a plausible cause for increased overall excitability and functional connectivity following TUS in a cortical circuit (Yaakub et al., 2023). Together, these findings underscore the intricate and multifaceted nature of the impact of mechanical *ARF* on neural systems.

**Figure 2.**
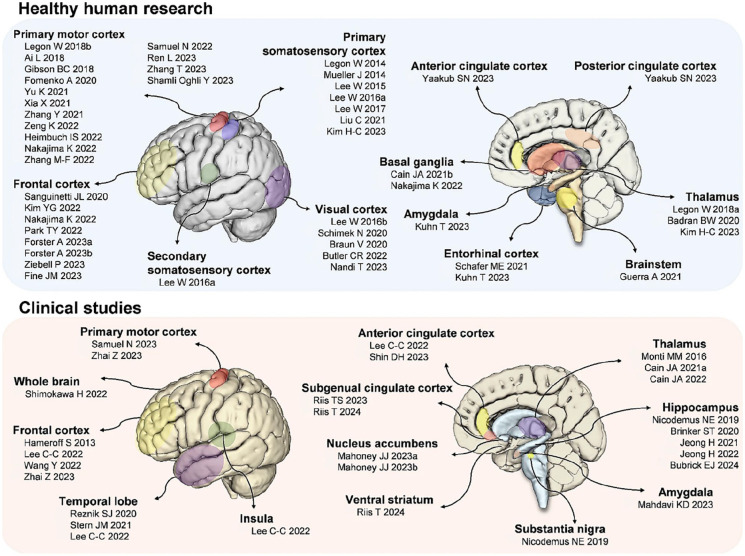
Overview of TUS stimulation studies in humans. Source: Lee et al. (2024).

Research in the field of TUS has predominantly focused on preclinical studies utilising rodents (Blackmore et al., 2023). When delving into the study of neuropsychiatric disorders that impact the PFC, non-human primates (NHPs) can be considered as a suitable animal model due to the human-like PFC in NHPs (Lear et al., 2022). The first TUS study in humans was conducted by Legon et al. in 2014, targeting the primary somatosensory cortex of healthy volunteers. This pioneering study employed a within-subjects, sham-controlled design, and the results indicated an increase in electroencephalogram (EEG) somatosensory evoked potentials (Legon et al., 2014). In addition, several studies have targeted the primary motor cortex (M1), visual cortex, thalamus, prefrontal cortex, anterior temporal lobe and hippocampus (Figure 2) (Blackmore et al., 2023; Butler et al., 2022; Kuhn et al., 2023; Lee et al., 2024; Sarica et al., 2022). Furthermore, TUS has been shown to induce changes in neuronal excitability, influencing the spontaneous firing rate of neurons which may impact cognitive functioning and behaviour long-term over several days or weeks (Bault et al., 2024; Darmani et al., 2022).

### TUS in psychiatry

Two early studies exploring the potential of targeting the fronto-temporal cortex with TUS to induce changes in mood and resting-state connectivity have yielded preliminary insights. While no changes in depression symptoms were found in students with elevated Beck’s Depression Inventory (BDI) scores (10–25), trait worry was reduced and functional connectivity in emotion-related networks changed following TUS (Reznik et al., 2020; Sanguinetti et al., 2020). Notably, the absence of an active control condition limits the ability to attribute these effects solely to TUS, highlighting the necessity for further research with more rigorous controls to substantiate these preliminary findings (Reznik et al., 2020). More recently, a study employing six repeated sessions of TUS over 2 weeks over the left dlPFC has shown improvements in Montgomery-Asberg Depression Rating Scale (MADRS) scores and associated functional connectivity changes (Oh et al., 2024). In addition, case studies have provided first evidence for TUS effects in TRD. For example, a patient receiving suppressive TUS targeting the SCC experienced remission of depressive symptoms within 24 h, with effects sustained for at least 6 weeks, accompanied by reduced fMRI-BOLD activation of the SCC (Riis et al., 2023). Similarly, anterior nucleus of the thalamus (ANT) stimulation reduced depressive symptoms and induced connectivity changes in another individual with TRD (Fan et al., 2024). Furthermore, in two TRD patients, TUS targeting the SCC and ventral striatum resulted in mood improvements over 6 weeks without any reported side effects (Riis et al., 2024).

For treatment-refractory anxiety disorders, a series of weekly TUS sessions applied to the right amygdala over 8 weeks significantly reduced anxiety, with 64% of participants reporting clinically meaningful improvements. However, again, the absence of a control group limits the interpretation of these findings (Mahdavi et al., 2023).

In patients with schizophrenia, repetitive excitatory TUS targeting the left dlPFC demonstrated a significant reduction in negative symptoms and improvements in cognitive performance during continuous performance tasks (Table 1; Zhai et al., 2023). Small, exploratory studies with individuals with SUDs demonstrated that bilateral TUS applied to the NAcc was associated with reduced cravings for various substances and reported mood enhancements persisting 90 days post-follow-up (Mahoney et al., 2023a, 2023b).

**Table 1.** Overview of TUS stimulation targets for psychiatric symptoms used in humans.

Target	Condition	*N*	Control	Outcomes	Details	Study
Amygdala	GAD	25	None	HAM-A BAI PGI-I (*offline*)	Weekly 10-min stimulation over 8 weeks resulted in clinically significant decrease in anxiety in 64% of patients.	Mahdavi et al. (2023)
Ventral Striatum	MDD	2	Active Sham	GASE (*offline*)	Single session led to no significant decrease in depression and anxiety scores in TRD patients.	Riis et al. (2024)
Temporal Cortex	MDD	24	Sham	VAMS BDI-II OASIS (*offline*)	Reduced trait worry and increased global affect after five sessions.	Reznik et al. (2020)
Subcallossal cingulate cortex (SCC)	MDD	1	Active Sham	fMRI (*online*) HDRS-6 (*offline*)	Decreased BOLD SCC signal and resolved symptoms in TRD patient for at least 6 weeks.	Riis et al. (2023)
		2	Active Sham	7-point scale mood ratings *(offline)*	Single session of at least 60 s led to sustained lowered depression and anxiety scores in TRD patients for at least 6 weeks.	Riis et al. (2024)
Anterior Nucleus of Thalamus (ANT)	MDD	1	Active Sham	fMRI (online) VAS HDRS-6 (offline)	Single session reduced VAS depression and suppressed default mode network connectivity; associated with improvement in depressive symptoms.	Fan et al. (2024)
M1	Schizophrenia	12	Sham	TMS-induced MEPs (*offline*)	Ipsilateral M1 MEPs increased for up to 15 min after stimulation, suggesting LTP plasticity modulation.	Zhai et al. (2023)
Nucleus Accumbens (NAcc)	SUD	5	Sham	Craving ratings (*offline*)	Bilateral stimulation led to decreases in craving and increases in mood.	Mahoney et al. (2023a, 2023b)
Dorsolateral Prefrontal Cortex (dlPFC)	Schizophrenia	13	Sham	SANS PANSS (*offline*)	15 sessions of excitatory repetitive stimulation relieved negative symptoms and improved cognitive performance.	Zhai et al. (2023)
	MDD	23	Sham	fMRI MADRS (*offline*)	3 weekly sessions over 2 weeks led to improved MADRS scores and higher functional connectivity between the subcallosal anterior cingulate cortex and bilateral prefrontal cortex, among others.	Oh et al. (2024).

GAD: generalised anxiety disorder; HAM-A: Hamilton Anxiety Rating Scale; BAI: Beck Anxiety Inventory; PGI-I: Patient Global Impression-Improvement; MDD: major depressive disorder; GASE: global assessment of side effects; TRD: treatment-resistant depression; VAMS: visual analogue mood scale; BDI-II: Beck Depression Inventory-II; OASIS: Overall Anxiety Severity and Impairment Scale; SCC: subcallosal cingulate cortex; HDRS-6: 6-item Hamilton Depression Rating Scale; ANT: anterior nucleus of the thalamus; MEPs: motor-evoked potentials; LTP: long-term potentiation; SUD: substance use disorder; SANS: Scale for the Assessment of Negative Symptoms; PANSS: Positive and Negative Syndrome Scale; MADRS: Montgomery-Asberg Depression Rating Scale; TUS: transcranial ultrasonic stimulation; fMRI: functional magnetic resonance imaging; M1: primary motor cortex; TMS: transcranial magnetic stimulation.

These findings, along with others, contribute to our understanding of the potential role of ultrasound in modulating emotion regulation and addressing psychiatric concerns (Arulpragasam et al., 2022). Taken together, they highlight the potential of TUS as a tool to improve symptom severity in psychiatric conditions. However, due to the preliminary and limited nature of the evidence, compounded by small sample sizes and lack of controls, further research is essential to rigorously establish the safety and efficacy of TUS in clinical settings.

**Table:** 

Practical considerations for TUS
Subjects should be informed about the theoretical risk of inducing seizures and individuals with a personal or family history of epilepsy must be excluded during screening (Murphy et al., 2025). Acoustic simulations are crucial for predicting transcranial wave travel, requiring measures to address air, refraction and reflection; a vital step involves using a hydrophone (underwater microphone) in a water tank to verify transducer functionality and ensure accurate property calculation and reporting (Klein et al., 2024). Concurrently, an RF Wattmeter can be used to verify the voltage and current emanating from the transducer. Ideally, a high-quality CT or pseudo-CT generated from an ultra-short MRI image is taken for each subject to improve trajectory planning. In the absence of gold-standard CT or intermediate pseudo-CT solutions, the maximum acoustic transmission can conservatively be estimated using a three-layer skull model (Attali et al., 2023). However, taking into account individual skull morphology during ultrasound planning can greatly enhance the precision of the trajectory, particularly for regions with irregular skull anatomy. MNI coordinates of the target area can then be identified and used in a trajectory planning tool. When planning the target, spatial constraints around ears, eyes and sinuses as well as off-target effects need to be taken into consideration (Aubry et al., 2023; Beisteiner et al., 2024; Murphy et al., 2025). Depth limitations for focal distance set by the transducer should also be considered. Throughout the process, biophysical safety guidelines set by ITRUSST should be followed (Aubry et al., 2023). Optimal trajectories should target the flatter regions of the skull and avoid sharp angles to prevent reflections and heating at the skull. Neuronavigation can be used for infrared optical navigation based on the planned trajectory and yields up to 2 mm accuracy, depending on the system employed. The so-called auditory confound, which is caused by the sound perceived due to the TUS signal envelope, remains a challenge (Ainslie Johnstone et al., 2021). The pitch and intensity of the sound vary based on the signal parameters (Braun et al., 2020; Johnstone et al., 2021). To mitigate this issue, studies should employ an active control procedure, ramp the ultrasound to reduce its audibility, utilise bone-conducting headphones with auditory masking, and include blinding questionnaires (Braun et al., 2020; Kop et al., 2024). Sensory features of the ultrasound (e.g. warmth, tingling) can only be mimicked with an active control.

TUS: transcranial ultrasonic stimulation; MRI: magnetic resonance imaging; ITRUSST: International Consortium for Transcranial Ultrasonic Stimulation Safety and Standards.

## Temporal Interference (TI) Stimulation

**Figure 3.**
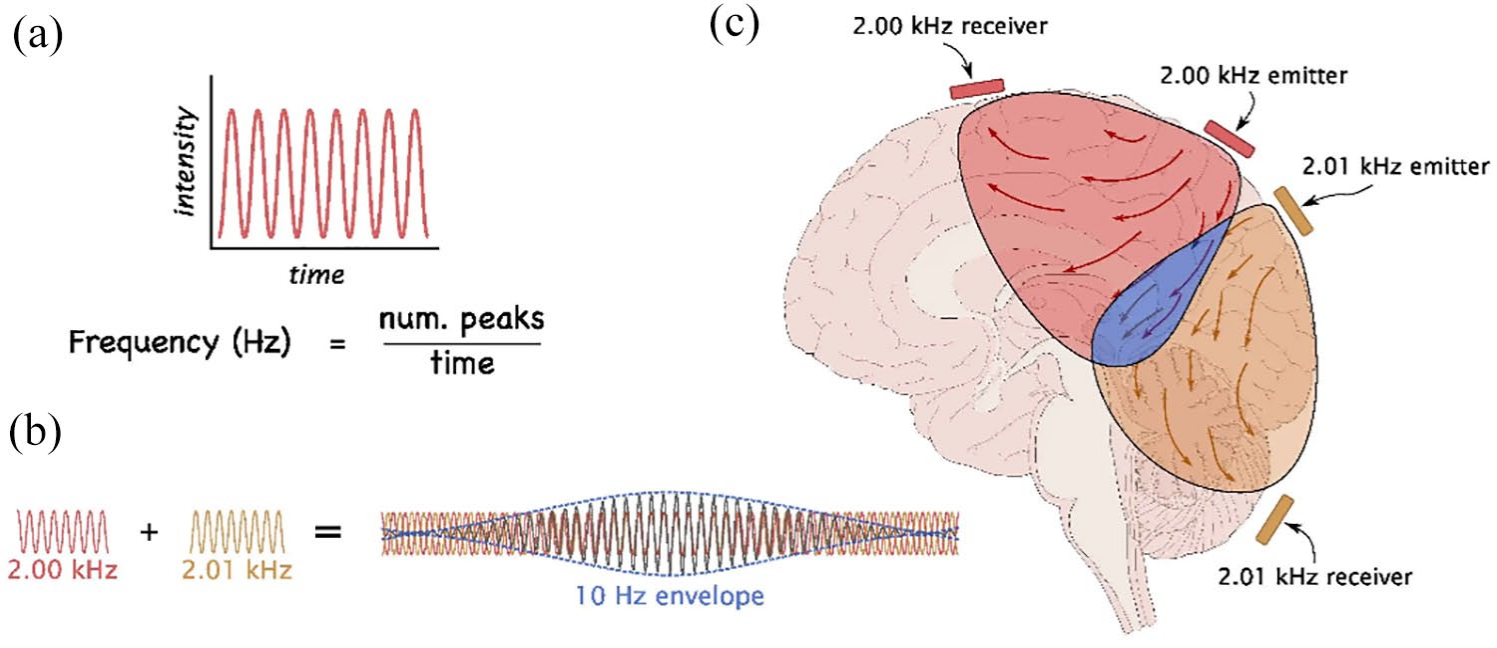
Schematic description of Temporal Interference Stimulation. (a) When two waves of distinct frequencies intersect, (b) they give rise to an envelope wave with a frequency equivalent to the difference between the original waves’ frequencies. For example, so-called carrier frequencies of 2.01 and 2.00 kHz result in 10 Hz. (c) When these fields are now applied to a brain (2.00 kHz in red and 2.01 kHz in orange), neuronal responses may be elicited within the resulting envelope field (10 Hz in blue), but not elsewhere. Source: Zhang and Utter (2018).

Another emerging NIBS method is TI, which is loosely based on *interferential current therapy* (*ICT*) developed in the 1950s. TI offers spatial specificity higher than tES but lower than TMS and TUS (Nasr et al., 2022; Zhu and Yin, 2023). It provides fewer peripheral stimulation confounds compared with tES. However, TI requires higher current for equivalent direct effects on neural activity as tACS (Nasr et al., 2022). In TI, two pairs of electrodes are placed on the scalp which deliver sinusoidal alternative currents at high frequencies (*HF*). By introducing a subtle *frequency shift* between two alternating *carrier currents*, an *oscillating amplitude-modulated (AM) envelope* emerges at the frequency difference between the two currents (Figure 3). For example, carrier frequencies of 2 and 2.01 kHz lead to modulation at 0.01 kHz (or 10 Hz) where the two currents meet in the brain, but not elsewhere (Grossman et al., 2017; Ma et al., 2021). This is based on the assumption that the carrier frequencies are too high to drive effective neural firing, thus ensuring the spatial specificity within the area of temporal interference in the envelope (Mirzakhalili et al., 2020). Neural activation in this region is driven by the low-frequency envelope of the interference pattern, aligning with the frequency-following properties of various neuronal populations (Caldas-Martinez et al., 2024). A recent pioneer study demonstrated the use of pulse-width modulated temporal interference (*PWM-TI*). In *PWM-TI* square and not sinusoidal waves are employed and the pulse-width is modulated rather than the amplitude itself (Luff et al., 2024). Overall, TI has been established to have higher spatial specificity than tACS and allows for the targeting of deeper regions of the brain (Nasr et al., 2022).

The potential of TI to selectively modulate neuronal activity within the envelope while sparing surrounding areas has been verified in computer models, rodents and, recently, humans (Esmaeilpour et al., 2021; Grossman et al., 2017; Violante et al., 2023; Yatsuda et al., 2024). The spatial specificity of TI hinges on the contrast in currents between the envelope in deep areas of the brain and the cortex as well as the sensitivity of neuronal networks to these electric fields (Esmaeilpour et al., 2021). In the first human validation study for TI, Violante et al. (2023) targeted the hippocampus in human cadavers and measured effects using implanted intracranial electrodes. The study revealed that the normalised envelope modulation amplitude (delivered at 5 Hz) was approximately 75% larger in the hippocampus compared with the surrounding cortex, further supporting the spatial specificity of TI. In a follow-up experiment on living humans, continuous TI stimulation during a face-naming task (self-paced, mean 44.53 ± 3-min stimulation) within the theta-band during memory encoding led to a reduction in the hippocampal BOLD signal measured by fMRI. However, this stimulation did not exhibit a significant impact on response type or reaction time in the mnemonic encoding and recall task (Violante et al., 2023). These findings suggest that theta-band TI stimulation may decrease metabolic demand in the hippocampus and impact memory function. This research signifies a significant step forward in understanding the intricacies of transcranial temporal interference stimulation and its potential implications for cognitive processes in humans (Violante et al., 2023). Another recent study by Wessel et al. investigated the effects of striatal neuromodulation using TI on motor learning behaviour. TI applied to the striatum of healthy participants at Theta-band frequency increased local neuronal activity and associated motor network measured by fMRI (Table 3). Furthermore, the stimulation improved motor performance in a sequential finger-tapping task (SFTT), particularly in older subjects (Wessel et al., 2023).

**Table 2.** Overview of TI stimulation targets for psychiatric symptoms used in humans.

Target	Condition	*N*	Control	Outcomes	Details	Study
Subgenual anterior cingulate cortex (sgACC)	MDD	30	Sham	RS-MRI (*offline*)	Ongoing pilot study with 10 sessions of stimulation to establish target engagement of the sgACC through resting-state MRI.	Demchenko et al. (2024)
Nucleus Accumbens (NAcc)	Bipolar disorder	36	None	HAMD QIDS MADRS HAMA THINC-it (*offline)*	Twice daily 20 min stimulation over 1 week led to reductions in symptom scores and improved memory and executive function.	Zhou et al. (2024)
Dorsolateral Prefrontal Cortex (dlPFC)	MDD	9	Sham	RS-fMRI HAMD-6 Emotional Stroop task (*offline*)	Preliminary data show that 20-min stimulation led to increase in functional connectivity between sgACC and DMN but no changes in in HAMD-6 scores.	Yan et al. (2024)

sgACC: subgenual anterior cingulate cortex; NAcc: nucleus accumbens; HAMD: Hamilton Depression Rating Scale; QIDS: Quick Inventory of Depressive Symptomatology; MADRS: Montgomery–Asberg Depression Rating Scale; HAMA: Hamilton Anxiety Rating Scale; THINC-it: Tool for Health Improvement through Neurocognitive Change; dlPFC: dorsolateral prefrontal cortex; MDD: major depressive disorder; RS-fMRI: Resting-State functional magnetic resonance imaging; DMN: Default Mode Network.

**Table 3.** Comparison of TUS and TI.

	TUS	TI
Precision	Millimetre-level spatial specificity, surpassing both tES and TMS; affected by skull properties and acoustic distortions (Bystritsky and Korb, 2015; Lee et al., 2024; Legon et al., 2020; Martin et al., 2024b; Nasr et al., 2022; Rabut et al., 2020).	Centimetre-level spatial specificity, surpassing tES but lower than TMS; precision depends on electric field amplitude modulation and neuronal sensitivity (Botzanowski et al., 2023; Esmaeilpour et al., 2021; Nasr et al., 2022; Violante et al., 2023).
Target depth	Effective for both subcortical and cortical regions, with no spatial restrictions. Previously targeted areas in studies include the hippocampus, thalamus, and nucleus accumbens. Uses acoustic frequencies between 250–700 kHz, allowing for better skull penetration (Lee et al., 2024).	Effective for both subcortical and cortical regions, with no spatial restrictions. Previously targeted areas in studies include the hippocampus and striatum. Deep targeting occurs due to the envelope electric field generated by interference (Liu et al., 2024; Violante et al., 2023; Zhu and Yin, 2023).
Target engagement	Can be directly observed through tissue displacement via MR-ARFI. Indirect observation methods include fMRI, EEG, PET and metabolic imaging techniques to assess functional, network-level and neurochemical changes. Long-term behavioural and cognitive effects, such as motor learning or mood regulation, have been demonstrated (Blackmore et al., 2023; Lee et al., 2024; Rabut et al., 2020; Yaakub et al., 2023).	Indirect observation methods include fMRI, EEG, PET and metabolic imaging techniques to assess functional, network-level and neurochemical changes. fMRI studies demonstrate localised BOLD signal changes and reduced hippocampal metabolic demand during memory tasks. Associated improvements in motor learning and memory encoding have been reported (Violante et al., 2023; Wessel et al., 2023).
Bio-mechanisms	Under active investigation; likely based on acoustic radiation force (ARF) acting on the neural lipid bilayer. Activates mechanosensitive ion channels (TRPP1/2, TRPC1, Piezo1) and alters membrane capacitance, resulting in neuronal excitability (Blackmore et al., 2023; Plaksin et al., 2014; Rabut et al., 2020; Ranade et al., 2015; Wahab et al., 2012).	Achieved through amplitude modulation of two high-frequency currents (e.g. 2.00 and 2.01 kHz) leading to an envelope electric field at the difference frequency (e.g. 10 Hz) (Grossman et al., 2017). This envelope selectively stimulates neurons in the target region by modulating transmembrane potentials, inducing depolarisation or hyperpolarisation (Mirzakhalili et al., 2020). TI may influence oscillatory brain activity by entraining specific neuronal circuits, thereby potentially altering synaptic plasticity and connectivity in targeted networks (Esmaeilpour et al., 2021; Ma et al., 2021).
Blinding	Audible sounds emitted during stimulation can disrupt blinding. Mitigation strategies include bone-conduction headphones, ramping of ultrasound, auditory masking and blinding questionnaires (Braun et al., 2020; Johnstone et al., 2021).	No unique challenges reported to date due to the use of electric fields, which do not generate acoustic artefacts (Zhang et al., 2022).
Safety	High-safety profile when adhering to established biophysical guidelines, with optimal transducer positioning and adherence to ITRUSST standards ensuring minimal risk of adverse effects (Aubry et al., 2023; Bystritsky and Korb, 2015; Legon et al., 2020; Murphy et al., 2025).	Considered safe with no major side effects apart from occasionally reported scalp discomfort (Piao et al., 2022; Vassiliadis et al., 2024; Zhu and Yin, 2023).
Ease of use	Moderate; requires precise trajectory planning using CT/MRI-based neuronavigation. Calibration of ultrasound parameters, such as intensity, frequency and pulse repetition, is essential to avoid off-target effects and skull heating (Aubry et al., 2023; Beisteiner et al., 2024; Bystritsky and Korb, 2015; Lee et al., 2024).	Moderate; MRI-based models are beneficial but not mandatory, allowing for simpler setups using common scalp landmarks. However, foregoing MRI introduces a trade-off, as subject-specific head models improve precision but add complexity and variability (Violante et al., 2023; Yatsuda et al., 2024).
Cost	Moderate; requires specialised ultrasound transducers, neuronavigation systems, and imaging equipment for skull-based calibration (Lee et al., 2024).	Relatively more cost-effective, as it primarily utilises standard electric field generators and adhesive electrodes, which are commonly available in many labs, potentially eliminating the need for additional equipment purchases (*TI Solutions*, n.d.).

TUS: transcranial ultrasonic stimulation; TI: temporal interference stimulation; tES: transcranial electrical stimulation; MR-ARFI: MR Acoustic Radiation Force Imaging; fMRI: functional magnetic resonance imaging; BOLD: blood-oxygen-level-dependent; GABA: gamma-aminobutyric acid; TMS: transcranial magnetic stimulation; ARF: acoustic radiation force; ITRUSST: International Consortium for Transcranial Ultrasonic Stimulation Safety and Standards.

### TI in psychiatry

Due to its novel nature, very little is known about potential effects of TI on affect and general psychopathology, with first studies being published just this past year. In a single-arm clinical trial targeting the left NAcc in patients with bipolar disorder, participants underwent 10, 20-min sessions over 1 week. The study reported preliminary evidence supporting the effectiveness of TI stimulation in alleviating depressive symptoms and enhancing cognitive function in these patients, however, lacked a control (Zhou et al., 2024). A double-blind sham-controlled study examined the effects of acute TIS applied to the left dlPFC in MDD, with preliminary results showing increased functional connectivity between the sgACC and default mode network (DMN) but no changes in depression scales (Yan et al., 2024). Furthermore, a recently published pilot study protocol details a double-blind, randomised, sham-controlled trial investigating the effects of repetitive TI stimulation on the sgACC in patients with MDD (Table 2) (Demchenko et al., 2024).

Targeting the amygdala, TI could be explored as a means to normalise hyperactive activity in MDD as well as exaggerated fear responses in PTSD and generalised anxiety disorder (GAD). The application might be most effective when combined with prolonged exposure therapy, working synergistically to alleviate the impact of heightened fear responses associated with these conditions. A large-scale fMRI study identified four possible depression subtypes based on distinct connectivity patterns, serving as potential biomarkers for diagnosis and guiding personalised stimulation therapy (Drysdale et al., 2017). Furthermore, given its role in reward processing and motivation, the ventral striatum could be targeted to treat individuals experiencing low affect such as anhedonia. To enhance its effectiveness, this approach could be paired with behavioural activation therapy for MDD, acknowledging the ventral striatum’s pivotal role in reward processing and motivation (Fani and Treadway, 2023). These avenues underscore the potential of TI for tailored interventions in individuals facing distinct psychiatric challenges which still require investigation and replication.

## Way forward

Recent advances in NIBS techniques such as TUS and TI enable researchers to investigate the pathophysiology of psychiatric symptoms at heightened precision, which could yield viable treatment protocols in clinical settings. This is especially imminent in psychiatric patient populations where a lack of treatment response and unsatisfactory remission rates are not uncommon. Furthermore, biomarkers could enhance personalised treatments by optimising stimulation parameters and identifying suitable patients. While their high spatial precision enables direct brain modulation, developing reliable biomarkers remains essential to monitor effects and improve outcomes among all NIBS (Cash and Zalesky, 2024; Murphy and Fouragnan, 2024). However, small sample sizes and lack of consistent control conditions in existing studies make it premature to draw definitive conclusions about the efficacy of these methods, underscoring the need for larger, well-controlled clinical trials. The question of whether these methods are viable treatments, and if so, how they may best be used to augment current therapies or as standalone treatments, has yet to be addressed by further clinical research. Previous studies combining brain stimulation techniques with therapy, such as rTMS with exposure therapy have shown promising results in anxiety disorders, suggesting that similar approaches with TUS and TI warrant exploration (Kan et al., 2020). TUS seems to have the highest spatial and potentially also mechanistic specificity among all NIBS methods and may be particularly useful in stimulating limbic structures. TI has similarly high mechanistic specificity yet is inferior in terms of its spatial specificity. Despite the benefits, practical considerations such as the need for MRI or CT scans, the complexity of the set-up, mobility and cost must also be considered. Common challenges of TUS including intricacies in target planning attributed to the acoustic properties of the skull, potential auditory confounds, and challenges in maintaining blinding, might be encountered to a lesser extent with TI.

In conclusion, the remarkable advancements in TUS and TI offer significant potential to refine our understanding of psychiatric symptoms and pave the way for developing more effective treatment strategies. However, their clinical translation is tempered by the scarce and heterogeneous landscape of existing studies, which underscores the need for a robust evidence base to validate their efficacy and safety. Addressing practical considerations such as cost, accessibility and technical complexities, while simultaneously overcoming inherent methodological challenges, will require innovative approaches and interdisciplinary collaboration. Only through these concerted efforts can we fully realise their promise and integrate these techniques effectively into clinical practice.

## References

[bibr1-23982128251322241] *Abbott MediaRoom* (n.d.) Abbott initiates clinical study to evaluate the use of its deep brain stimulation system to manage severe depression. Available at: https://abbott.mediaroom.com/2024-09-04-Abbott-Initiates-Clinical-Study-to-Evaluate-the-Use-of-Its-Deep-Brain-Stimulation-System-to-Manage-Severe-Depression (accessed 14 December 2024).

[bibr2-23982128251322241] Alagapan S Choi KS Heisig S , et al (2023) Cingulate dynamics track depression recovery with deep brain stimulation. Nature 622(7981): 130–138. 37730990 10.1038/s41586-023-06541-3 PMC10550829

[bibr3-23982128251322241] Amad A Fovet T (2021) rTMS for depression: The difficult transition from research to clinical practice. Australian & New Zealand Journal of Psychiatry 56(1): 14–15. 33982627 10.1177/00048674211011242

[bibr4-23982128251322241] Arulpragasam AR Van’t Wout-Frank M Barredo J , et al (2022) Low intensity focused ultrasound for non-invasive and reversible deep brain neuromodulation – A paradigm shift in psychiatric research. Frontiers in Psychiatry 13: 825802. 35280168 10.3389/fpsyt.2022.825802 PMC8907584

[bibr5-23982128251322241] Asir B Boscutti A Fenoy AJ , et al (2024) Deep Brain Stimulation (DBS) in Treatment-Resistant Depression (TRD): Hope and concern. Advances in Experimental Medicine and Biology 1456: 161–186. 39261429 10.1007/978-981-97-4402-2_9

[bibr6-23982128251322241] Attali D Tiennot T Schafer M , et al (2023) Three-layer model with absorption for conservative estimation of the maximum acoustic transmission coefficient through the human skull for transcranial ultrasound stimulation. Brain Stimulation 16(1): 48–55. 36549480 10.1016/j.brs.2022.12.005

[bibr7-23982128251322241] Aubry J-F Attali D Schafer M , et al (2023) ITRUSST consensus on biophysical safety for transcranial ultrasonic stimulation. Available at: http://arxiv.org/abs/2311.05359 (accessed 30 April 2024). 10.1016/j.brs.2025.10.007 PMC12644226 41072763

[bibr8-23982128251322241] Aust S Brakemeier E-L Spies J , et al (2022) Efficacy of augmentation of cognitive behavioral therapy with transcranial direct current stimulation for depression: A randomized clinical trial. JAMA Psychiatry 79(6): 528–537. 35442431 10.1001/jamapsychiatry.2022.0696 PMC9021985

[bibr9-23982128251322241] Bault N Yaakub SN Fouragnan E (2024) Early-phase neuroplasticity induced by offline transcranial ultrasound stimulation in primates. Current Opinion in Behavioral Sciences 56: 101370.

[bibr10-23982128251322241] Beisteiner R Lozano A Di Lazzaro V , et al (2024) Clinical recommendations for non-invasive ultrasound neuromodulation. Brain Stimulation 17(4): 890–895. 39084519 10.1016/j.brs.2024.07.013

[bibr11-23982128251322241] Bergmann TO Hartwigsen G (2021) Inferring causality from noninvasive brain stimulation in cognitive neuroscience. Journal of Cognitive Neuroscience 33(2): 195–225. 32530381 10.1162/jocn_a_01591

[bibr12-23982128251322241] Blackmore DG Razansky D Götz J (2023) Ultrasound as a versatile tool for short- and long-term improvement and monitoring of brain function. Neuron 111(8): 1174–1190. 36917978 10.1016/j.neuron.2023.02.018

[bibr13-23982128251322241] Botzanowski B Acerbo E Lehmann S , et al (2023) Focal control of non-invasive deep brain stimulation using multipolar temporal interference. bioRxiv. Available at: http://biorxiv.org/content/early/2024/08/26/2023.09.05.556444.abstract 10.1186/s42234-025-00169-6 PMC11948895 40140933

[bibr14-23982128251322241] Bourla A Chaneac E Poulet E , et al (2020) Acceptability, attitudes and knowledge towards Transcranial Magnetic Stimulation (TMS) among psychiatrists in France. L’Encephale 46(2): 88–95. 10.1016/j.encep.2019.07.003 31522836

[bibr15-23982128251322241] Braun V Blackmore J Cleveland RO , et al (2020) Transcranial ultrasound stimulation in humans is associated with an auditory confound that can be effectively masked. Brain Stimulation 13(6): 1527–1534. 32891872 10.1016/j.brs.2020.08.014 PMC7710976

[bibr16-23982128251322241] Brunoni AR Moffa AH Fregni F , et al (2016) Transcranial direct current stimulation for acute major depressive episodes: Meta-analysis of individual patient data. The British Journal of Psychiatry 208(6): 522–531. 27056623 10.1192/bjp.bp.115.164715 PMC4887722

[bibr17-23982128251322241] Butler CR Rhodes E Blackmore J , et al (2022) Transcranial ultrasound stimulation to human middle temporal complex improves visual motion detection and modulates electrophysiological responses. Brain Stimulation 15(5): 1236–1245. 36067978 10.1016/j.brs.2022.08.022

[bibr18-23982128251322241] Bystritsky A Korb AS (2015) A review of low-intensity transcranial focused ultrasound for clinical applications. Current Behavioral Neuroscience Reports 2(2): 60–66.

[bibr19-23982128251322241] Caldas-Martinez S Goswami C Forssell M , et al (2024) Cell-specific effects of temporal interference stimulation on cortical function. Communications Biology 7(1): 1076. 10.1038/s42003-024-06728-y PMC11369164 39223260

[bibr20-23982128251322241] Cash RFH Zalesky A (2024) Personalized and circuit-based transcranial magnetic stimulation: Evidence, controversies, and opportunities. Biological Psychiatry 95(6): 510–522. 38040047 10.1016/j.biopsych.2023.11.013

[bibr21-23982128251322241] Center for Devices and Radiological Health (CDRH) (2023) Marketing Clearance of Diagnostic Ultrasound Systems and Transducers. FDA. Available at: https://www.fda.gov/regulatory-information/search-fda-guidance-documents/marketing-clearance-diagnostic-ultrasound-systems-and-transducers (accessed 23 February 2024).

[bibr22-23982128251322241] Chan JL Carpentier AV Middlebrooks EH , et al (2024) Current perspectives on tractography-guided deep brain stimulation for the treatment of mood disorders. Expert Review of Neurotherapeutics 24(1): 11–24. 38037329 10.1080/14737175.2023.2289573

[bibr23-23982128251322241] Chen L Klooster DCW Tik M , et al (2023) Accelerated repetitive transcranial magnetic stimulation to treat major depression: The past, present, and future. Harvard Review of Psychiatry 31(3): 142–161. 37171474 10.1097/HRP.0000000000000364 PMC10188211

[bibr24-23982128251322241] Clinicaltrials.gov (n.d.) Available at: https://clinicaltrials.gov/study/NCT06423430 (accessed 14 December 2024).

[bibr25-23982128251322241] Cole EJ O’Sullivan SJ Tik M , et al (2024) Accelerated theta burst stimulation: Safety, efficacy, and future advancements. Biological Psychiatry 95(6): 523–535. 38383091 10.1016/j.biopsych.2023.12.004 PMC10952126

[bibr26-23982128251322241] Cole EJ Phillips AL Bentzley BS , et al (2022) Stanford neuromodulation therapy (SNT): A double-blind randomized controlled trial. The American Journal of Psychiatry 179(2): 132–141. 34711062 10.1176/appi.ajp.2021.20101429

[bibr27-23982128251322241] Darmani G Bergmann TO Butts Pauly K , et al (2022) Non-invasive transcranial ultrasound stimulation for neuromodulation. Clinical Neurophysiology 135: 51–73. 35033772 10.1016/j.clinph.2021.12.010

[bibr28-23982128251322241] Davidson B Bhattacharya A Sarica C , et al (2024) Neuromodulation techniques – From non-invasive brain stimulation to deep brain stimulation. Neurotherapeutics 21(3): e00330. 10.1016/j.neurot.2024.e00330 PMC11103220 38340524

[bibr29-23982128251322241] Delaloye S Holtzheimer PE (2014) Deep brain stimulation in the treatment of depression. Dialogues in Clinical Neuroscience 16(1): 83–91. 24733973 10.31887/DCNS.2014.16.1/sdelaloye PMC3984894

[bibr30-23982128251322241] Demchenko I Rampersad S Datta A , et al (2024) Target engagement of the subgenual anterior cingulate cortex with transcranial temporal interference stimulation in major depressive disorder: A protocol for a randomized sham-controlled trial. Frontiers in Neuroscience 18: 1390250. 39268031 10.3389/fnins.2024.1390250 PMC11390435

[bibr31-23982128251322241] Di Biase L Falato E Di Lazzaro V (2019) Transcranial Focused Ultrasound (tFUS) and Transcranial Unfocused Ultrasound (tUS) neuromodulation: From theoretical principles to stimulation practices. Frontiers in Neurology 10: 549. 31244747 10.3389/fneur.2019.00549 PMC6579808

[bibr32-23982128251322241] Dougherty DD Rezai AR Carpenter LL , et al (2015) A randomized sham-controlled trial of deep brain stimulation of the ventral capsule/ventral striatum for chronic treatment-resistant depression. Biological Psychiatry 78(4): 240–248. 25726497 10.1016/j.biopsych.2014.11.023

[bibr33-23982128251322241] Drysdale AT Grosenick L Downar J , et al (2017) Resting-state connectivity biomarkers define neurophysiological subtypes of depression. Nature Medicine 23(1): 28–38. 10.1038/nm.4246 PMC5624035 27918562

[bibr34-23982128251322241] Esmaeilpour Z Kronberg G Reato D , et al (2021) Temporal interference stimulation targets deep brain regions by modulating neural oscillations. Brain Stimulation 14(1): 55–65. 33186778 10.1016/j.brs.2020.11.007 PMC9382891

[bibr35-23982128251322241] Fan JM Woodworth K Murphy KR , et al (2024) Thalamic transcranial ultrasound stimulation in treatment resistant depression. Brain Stimulation 17(5): 1001–1004. 39173737 10.1016/j.brs.2024.08.006 PMC11531731

[bibr36-23982128251322241] Fani N Treadway MT (2023) Potential applications of temporal interference deep brain stimulation for the treatment of transdiagnostic conditions in psychiatry. Neuropsychopharmacology 49(1): 305–306. 10.1038/s41386-023-01682-5 PMC10700552 37524751

[bibr37-23982128251322241] Fitzpatrick JM West JB (2001) The distribution of target registration error in rigid-body point-based registration. IEEE Transactions on Medical Imaging 20(9): 917–927. 11585208 10.1109/42.952729

[bibr38-23982128251322241] Fox MD Buckner RL White MP , et al (2012) Efficacy of transcranial magnetic stimulation targets for depression is related to intrinsic functional connectivity with the subgenual cingulate. Biological Psychiatry 72(7): 595–603. 22658708 10.1016/j.biopsych.2012.04.028 PMC4120275

[bibr39-23982128251322241] Friedrich MJ (2017) Depression is the leading cause of disability around the world. JAMA: The Journal of the American Medical Association 317(15): 1517. 10.1001/jama.2017.3826 28418490

[bibr40-23982128251322241] Gadot R Li N Shofty B , et al (2023) Tractography-based modeling explains treatment outcomes in patients undergoing deep brain stimulation for obsessive-compulsive disorder. Biological Psychiatry 96(2): 95–100. 36948900 10.1016/j.biopsych.2023.01.017 PMC10387502

[bibr41-23982128251322241] Gazdag G Ungvari GS (2019) Electroconvulsive therapy: 80 years old and still going strong. World Journal of Psychiatry 9(1): 1–6. 30631748 10.5498/wjp.v9.i1.1 PMC6323557

[bibr42-23982128251322241] Graat I Figee M Denys D (2017) The application of deep brain stimulation in the treatment of psychiatric disorders. International Review of Psychiatry 29(2): 178–190. 28523977 10.1080/09540261.2017.1282439

[bibr43-23982128251322241] Grosshagauer S Woletz M Vasileiadi M , et al (2024) Chronometric TMS-fMRI of personalized left dorsolateral prefrontal target reveals state-dependency of subgenual anterior cingulate cortex effects. Molecular Psychiatry 29(9): 2678–2688. 38532009 10.1038/s41380-024-02535-3 PMC11420068

[bibr44-23982128251322241] Grossman N Bono D Dedic N , et al (2017) Noninvasive deep brain stimulation via temporally interfering electric fields. Cell 169(6): 1029.e16–1041.e16. 10.1016/j.cell.2017.05.024 PMC5520675 28575667

[bibr45-23982128251322241] Holtzheimer PE Husain MM Lisanby SH , et al (2017) Subcallosal cingulate deep brain stimulation for treatment-resistant depression: A multisite, randomised, sham-controlled trial. The Lancet Psychiatry 4(11): 839–849. 28988904 10.1016/S2215-0366(17)30371-1

[bibr46-23982128251322241] Ironside M Browning M Ansari TL , et al (2019) Effect of prefrontal cortex stimulation on regulation of amygdala response to threat in individuals with trait anxiety: A randomized clinical trial. JAMA Psychiatry 76(1): 71–78. 30347011 10.1001/jamapsychiatry.2018.2172 PMC6583758

[bibr47-23982128251322241] Jing B Strassle Rojas S Lindsey BD (2023) Effect of skull porosity on ultrasound transmission and wave mode conversion at large incidence angles. Medical Physics 50(5): 3092–3102. 36810723 10.1002/mp.16318

[bibr48-23982128251322241] Johnson KA Okun MS Scangos KW , et al (2024) Deep brain stimulation for refractory major depressive disorder: A comprehensive review. Molecular Psychiatry 29(4): 1075–1087. 38287101 10.1038/s41380-023-02394-4 PMC11348289

[bibr49-23982128251322241] Johnstone A Nandi T Martin E , et al (2021) A range of pulses commonly used for human transcranial ultrasound stimulation are clearly audible. Brain Stimulation 14(5): 1353–1355. 34481096 10.1016/j.brs.2021.08.015

[bibr50-23982128251322241] Jung NY Rachmilevitch I Sibiger O , et al (2019) Factors related to successful energy transmission of focused ultrasound through a skull: A study in human cadavers and its comparison with clinical experiences. Journal of Korean Neurosurgical Society 62(6): 712–722. 31142101 10.3340/jkns.2018.0226 PMC6835146

[bibr51-23982128251322241] Kamel LY Xiong W Gott BM , et al (2022) Vagus nerve stimulation: An update on a novel treatment for treatment-resistant depression. Journal of the Neurological Sciences 434: 120171. 35158102 10.1016/j.jns.2022.120171

[bibr52-23982128251322241] Kan RLD Zhang BBB Zhang JJQ , et al (2020) Non-invasive brain stimulation for posttraumatic stress disorder: A systematic review and meta-analysis. Translational Psychiatry 10(1): 168. 32467579 10.1038/s41398-020-0851-5 PMC7256039

[bibr53-23982128251322241] Klein-Flügge MC Fouragnan EF Martin E (2024) The importance of acoustic output measurement and monitoring for the replicability of transcranial ultrasonic stimulation studies. Brain Stimulation 17(1): 32–34. 38092243 10.1016/j.brs.2023.12.002 PMC7618972

[bibr54-23982128251322241] Kop BR Oghli YS Grippe TC , et al (2024) Auditory confounds can drive online effects of transcranial ultrasonic stimulation in humans. Available at: https://dx.doi.org/10.7554/elife.88762.2 10.7554/eLife.88762 PMC11349300 39190585

[bibr55-23982128251322241] Kuhn T Spivak NM Dang BH , et al (2023) Transcranial focused ultrasound selectively increases perfusion and modulates functional connectivity of deep brain regions in humans. Frontiers in Neural Circuits 17: 1120410. 37091318 10.3389/fncir.2023.1120410 PMC10114286

[bibr56-23982128251322241] Lear A Baker SN Clarke HF , et al (2022) Understanding them to understand ourselves: The importance of NHP research for translational neuroscience. Current Research in Neurobiology 3: 100049. 36518342 10.1016/j.crneur.2022.100049 PMC9743051

[bibr57-23982128251322241] Lee K Park TY Lee W , et al (2024) A review of functional neuromodulation in humans using low-intensity transcranial focused ultrasound. Biomedical Engineering Letters 14(3): 407–438. 38645585 10.1007/s13534-024-00369-0 PMC11026350

[bibr58-23982128251322241] Lee W Weisholtz DS Strangman GE , et al (2021) Safety review and perspectives of transcranial focused ultrasound brain stimulation. Brain & Neurorehabilitation 14(1): e4. 10.12786/bn.2021.14.e4 PMC9879416 36742103

[bibr59-23982128251322241] Legon W Adams S Bansal P , et al (2020) A retrospective qualitative report of symptoms and safety from transcranial focused ultrasound for neuromodulation in humans. Scientific Reports 10(1): 5573. 32221350 10.1038/s41598-020-62265-8 PMC7101402

[bibr60-23982128251322241] Legon W Sato TF Opitz A , et al (2014) Transcranial focused ultrasound modulates the activity of primary somatosensory cortex in humans. Nature Neuroscience 17(2): 322–329. 24413698 10.1038/nn.3620

[bibr61-23982128251322241] Liston C Chen AC Zebley BD , et al (2014) Default mode network mechanisms of transcranial magnetic stimulation in depression. Biological Psychiatry 76(7): 517–526. 24629537 10.1016/j.biopsych.2014.01.023 PMC4209727

[bibr62-23982128251322241] Liu R Zhu G Wu Z , et al (2024) Temporal interference stimulation targets deep primate brain. NeuroImage 291: 120581. 38508293 10.1016/j.neuroimage.2024.120581

[bibr63-23982128251322241] Loo CK Husain MM McDonald WM , et al (2018) International randomized-controlled trial of transcranial Direct Current Stimulation in depression. Brain Stimulation 11(1): 125–133. 29111077 10.1016/j.brs.2017.10.011

[bibr64-23982128251322241] Lord B Sanguinetti JL Ruiz L , et al (2024) Transcranial focused ultrasound to the posterior cingulate cortex modulates default mode network and subjective experience: An fMRI pilot study. Frontiers in Human Neuroscience 18: 1392199. 38895168 10.3389/fnhum.2024.1392199 PMC11184145

[bibr65-23982128251322241] Lozano AM Lipsman N (2013) Probing and regulating dysfunctional circuits using deep brain stimulation. Neuron 77(3): 406–424. 23395370 10.1016/j.neuron.2013.01.020

[bibr66-23982128251322241] Luff CE Dzialecka P Acerbo E , et al (2024) Pulse-width modulated temporal interference (PWM-TI) brain stimulation. Brain Stimulation 17(1): 92–103. 38145754 10.1016/j.brs.2023.12.010

[bibr67-23982128251322241] Ma R Xia X Zhang W , et al (2021) High gamma and beta temporal interference stimulation in the human motor cortex improves motor functions. Frontiers in Neuroscience 15: 800436. 35046771 10.3389/fnins.2021.800436 PMC8761631

[bibr68-23982128251322241] Mahdavi KD Jordan SE Jordan KG , et al (2023) A pilot study of low-intensity focused ultrasound for treatment-resistant generalized anxiety disorder. Journal of Psychiatric Research 168: 125–132. 39491902 10.1016/j.jpsychires.2023.10.039

[bibr69-23982128251322241] Mahoney JJ Haut MW Carpenter J , et al (2023a) Low-intensity focused ultrasound targeting the nucleus accumbens as a potential treatment for substance use disorder: Safety and feasibility clinical trial. Frontiers in Psychiatry 14: 1211566. 37779628 10.3389/fpsyt.2023.1211566 PMC10540197

[bibr70-23982128251322241] Mahoney JJ III Thompson-Lake DGY Ranjan M , et al (2023b) Low-intensity focused ultrasound targeting the bilateral nucleus accumbens as a potential treatment for substance use disorder: A first-in-human report. Biological Psychiatry 94(11): e41–e43. 10.1016/j.biopsych.2023.06.031 37610405

[bibr71-23982128251322241] Martin E Aubry J-F Schafer M , et al (2024a) ITRUSST consensus on standardised reporting for transcranial ultrasound stimulation. Available at: https://www.ncbi.nlm.nih.gov/pubmed/38410648 10.1016/j.brs.2024.04.013 PMC12436198 38670224

[bibr72-23982128251322241] Martin E Roberts M Grigoras IF , et al (2024b) Ultrasound system for precise neuromodulation of human deep brain circuits. bioRxiv. Available at: http://biorxiv.org/content/early/2024/06/08/2024.06.08.597305.abstract 10.1038/s41467-025-63020-1 PMC12413462 40913042

[bibr73-23982128251322241] Marx W Penninx BWJH Solmi M , et al (2023) Major depressive disorder. Nature Reviews Disease Primers 9(1): 44. 10.1038/s41572-023-00454-1 37620370

[bibr74-23982128251322241] McClintock SM Martin DM Lisanby SH , et al (2020) Neurocognitive effects of transcranial direct current stimulation (tDCS) in unipolar and bipolar depression: Findings from an international randomized controlled trial. Depression and Anxiety 37(3): 261–272. 31944487 10.1002/da.22988

[bibr75-23982128251322241] McClintock SM Reti IM Carpenter LL , et al (2018) Consensus recommendations for the clinical application of repetitive transcranial magnetic stimulation (rTMS) in the treatment of depression. The Journal of Clinical Psychiatry 79(1): 16cs10905. 10.4088/JCP.16cs10905 PMC5846193 28541649

[bibr76-23982128251322241] *Mental Disorders* (2023) WHO. Available at: https://www.who.int/news-room/fact-sheets/detail/mental-disorders (accessed 20 November 2023).

[bibr77-23982128251322241] Menz MD Ye P Firouzi K , et al (2019) Radiation force as a physical mechanism for ultrasonic neurostimulation of the ex vivo retina. The Journal of Neuroscience 39(32): 6251–6264. 31196935 10.1523/JNEUROSCI.2394-18.2019 PMC6687898

[bibr78-23982128251322241] Meyer GM Hollunder B Li N , et al (2024) Deep brain stimulation for obsessive-compulsive disorder: Optimal stimulation sites. Biological Psychiatry 96(2): 101–113. 38141909 10.1016/j.biopsych.2023.12.010 PMC11190041

[bibr79-23982128251322241] Mirzakhalili E Barra B Capogrosso M , et al (2020) Biophysics of temporal interference stimulation. Cell Systems 11(6): 557.e5–572.e5. 10.1016/j.cels.2020.10.004 33157010

[bibr80-23982128251322241] Morriss R Briley PM Webster L , et al (2024) Connectivity-guided intermittent theta burst versus repetitive transcranial magnetic stimulation for treatment-resistant depression: A randomized controlled trial. Nature Medicine 30(2): 403–413. 10.1038/s41591-023-02764-z PMC10878976 38228914

[bibr81-23982128251322241] Murphy KR Fouragnan E (2024) The future of transcranial ultrasound as a precision brain interface. PLoS Biology 22(10): e3002884. 10.1371/journal.pbio.3002884 PMC11521279 39471185

[bibr82-23982128251322241] Murphy KR Farrell JS Gomez JL , et al (2022) A tool for monitoring cell type-specific focused ultrasound neuromodulation and control of chronic epilepsy. Proceedings of the National Academy of Sciences of the United States of America 119(46): e2206828119. 10.1073/pnas.2206828119 PMC9674244 36343238

[bibr83-23982128251322241] Murphy KR Nandi T Kop B , et al. (2025) A practical guide to transcranial ultrasonic stimulation from the IFCN-endorsed ITRUSST consortium. Clinical Neurophysiology 171: 192–226. Available at: https://doi.org/10.1016/j.clinph.2025.01.004 39933226 10.1016/j.clinph.2025.01.004

[bibr84-23982128251322241] Nasr K Haslacher D Dayan E , et al (2022) Breaking the boundaries of interacting with the human brain using adaptive closed-loop stimulation. Progress in Neurobiology 216: 102311. 35750290 10.1016/j.pneurobio.2022.102311

[bibr85-23982128251322241] National Institute for Health and Care Excellence (NICE) (2009) Overview – Guidance on the Use of Electroconvulsive Therapy: Guidance. London: NICE.

[bibr86-23982128251322241] Newman M Rasiah PK Kusunose J , et al (2024) Ultrasound modulates calcium activity in cultured neurons, glial cells, endothelial cells and pericytes. Ultrasound in Medicine & Biology 50(3): 341–351. 38087717 10.1016/j.ultrasmedbio.2023.11.004

[bibr87-23982128251322241] Office of the Commissioner (2020) FDA permits marketing of transcranial magnetic stimulation for treatment of obsessive compulsive disorder. US Food and Drug Administration (FDA). Available at: https://www.fda.gov/news-events/press-announcements/fda-permits-marketing-transcranial-magnetic-stimulation-treatment-obsessive-compulsive-disorder (accessed 22 February 2024).

[bibr88-23982128251322241] Oh J Ryu JS Kim J , et al (2024) Effect of low-intensity transcranial focused ultrasound stimulation in patients with major depressive disorder: A randomized, double-blind, sham-controlled clinical trial. Psychiatry Investigation 21(8): 885–896. 39111747 10.30773/pi.2024.0016 PMC11321877

[bibr89-23982128251322241] O’Reardon JP Cristancho P Peshek AD (2006) Vagus Nerve Stimulation (VNS) and treatment of depression: To the brainstem and beyond. Psychiatry 3(5): 54–63. 21103178 PMC2990624

[bibr90-23982128251322241] Pasquinelli C Hanson LG Siebner HR , et al (2019) Safety of transcranial focused ultrasound stimulation: A systematic review of the state of knowledge from both human and animal studies. Brain Stimulation 12(6): 1367–1380. 31401074 10.1016/j.brs.2019.07.024

[bibr91-23982128251322241] Pellow C Pichardo S Pike GB (2024) A systematic review of preclinical and clinical transcranial ultrasound neuromodulation and opportunities for functional connectomics. Brain Stimulation 17(4): 734–751. 38880207 10.1016/j.brs.2024.06.005

[bibr92-23982128251322241] Piao Y Ma R Weng Y , et al (2022) Safety evaluation of employing temporal interference transcranial alternating current stimulation in human studies. Brain Sciences 12(9): 1194. 36138930 10.3390/brainsci12091194 PMC9496688

[bibr93-23982128251322241] Pinton G Aubry JF Bossy E , et al (2012) Attenuation, scattering, and absorption of ultrasound in the skull bone. Medical Physics 39(1): 299–307. 22225300 10.1118/1.3668316

[bibr94-23982128251322241] Plaksin M Shoham S Kimmel E (2014) Intramembrane cavitation as a predictive bio-piezoelectric mechanism for ultrasonic brain stimulation. Physical Review X 4(1): 011004.

[bibr95-23982128251322241] Qu L Ge S Li N , et al (2019) Clinical evaluation of deep brain stimulation of nucleus accumbens/anterior limb of internal capsule for opioid relapse prevention: Protocol of a multicentre, prospective and double-blinded study. BMJ Open 9(2): e023516. 10.1136/bmjopen-2018-023516 PMC6398661 30765398

[bibr96-23982128251322241] Rabut C Yoo S Hurt RC , et al (2020) Ultrasound technologies for imaging and modulating neural activity. Neuron 108(1): 93–110. 33058769 10.1016/j.neuron.2020.09.003 PMC7577369

[bibr97-23982128251322241] Rachid F (2018) Maintenance repetitive transcranial magnetic stimulation (rTMS) for relapse prevention in with depression: A review. Psychiatry Research 262: 363–372. 28951141 10.1016/j.psychres.2017.09.009

[bibr98-23982128251322241] Ranade SS Syeda R Patapoutian A (2015) Mechanically activated ion channels. Neuron 87(6): 1162–1179. 26402601 10.1016/j.neuron.2015.08.032 PMC4582600

[bibr99-23982128251322241] Reed T Cohen Kadosh R (2018) Transcranial electrical stimulation (tES) mechanisms and its effects on cortical excitability and connectivity. Journal of Inherited Metabolic Disease 41(6): 1123–1130. 30006770 10.1007/s10545-018-0181-4 PMC6326965

[bibr100-23982128251322241] Reznik SJ Sanguinetti JL Tyler WJ , et al (2020) A double-blind pilot study of transcranial ultrasound (TUS) as a five-day intervention: TUS mitigates worry among depressed participants. Neurology, Psychiatry and Brain Research 37(6): 60–66.

[bibr101-23982128251322241] Riis TS Feldman DA Losser AJ , et al (2024) Device for multifocal delivery of ultrasound into deep brain regions in humans. IEEE Transactions on Biomedical Engineering 71(2): 660–668. 37695955 10.1109/TBME.2023.3313987 PMC10803076

[bibr102-23982128251322241] Riis TS Feldman DA Vonesh LC , et al (2023) Durable effects of deep brain ultrasonic neuromodulation on major depression: A case report. Journal of Medical Case Reports 17(1): 449. 37891643 10.1186/s13256-023-04194-4 PMC10612153

[bibr103-23982128251322241] Sanguinetti JL Hameroff S Smith EE , et al (2020) Transcranial focused ultrasound to the right prefrontal cortex improves mood and alters functional connectivity in humans. Frontiers in Human Neuroscience 14: 52. 32184714 10.3389/fnhum.2020.00052 PMC7058635

[bibr104-23982128251322241] Sarica C Nankoo JF Fomenko A , et al (2022) Human Studies of Transcranial Ultrasound neuromodulation: A systematic review of effectiveness and safety. Brain Stimulation 15(3): 737–746. 35533835 10.1016/j.brs.2022.05.002

[bibr105-23982128251322241] Shi R Wang Z Yang D , et al (2024) Short-term and long-term efficacy of accelerated transcranial magnetic stimulation for depression: A systematic review and meta-analysis. BMC Psychiatry 24(1): 109. 38326789 10.1186/s12888-024-05545-1 PMC10851556

[bibr106-23982128251322241] Sobstyl M Kupryjaniuk A Prokopienko M , et al (2022) Subcallosal cingulate cortex deep brain stimulation for treatment-resistant depression: A systematic review. Frontiers in Neurology 13: 780481. 35432155 10.3389/fneur.2022.780481 PMC9012165

[bibr107-23982128251322241] Tao Y Liang Q Zhang F , et al (2024) Efficacy of non-invasive brain stimulation combined with antidepressant medications for depression: A systematic review and meta-analysis of randomized controlled trials. Systematic Reviews 13(1): 92. 38509623 10.1186/s13643-024-02480-w PMC10953221

[bibr108-23982128251322241] *TI Solutions* (n.d.) TI Solutions. Available at: https://temporalinterference.com/ (accessed 2 December 2024).

[bibr109-23982128251322241] Tufail Y Matyushov A Baldwin N , et al (2010) Transcranial pulsed ultrasound stimulates intact brain circuits. Neuron 66(5): 681–694. 20547127 10.1016/j.neuron.2010.05.008

[bibr110-23982128251322241] Tyler WJ Tufail Y Finsterwald M , et al (2008) Remote excitation of neuronal circuits using low-intensity, low-frequency ultrasound. PLoS One 3(10): e3511. 10.1371/journal.pone.0003511 PMC2568804 18958151

[bibr111-23982128251322241] Vassiliadis P Stiennon E Windel F , et al (2024) Safety, tolerability and blinding efficiency of non-invasive deep transcranial temporal interference stimulation: First experience from more than 250 sessions. Journal of Neural Engineering 21(2): 024001. 10.1088/1741-2552/ad2d32 38408385

[bibr112-23982128251322241] Violante IR Alania K Cassarà AM , et al (2023) Non-invasive temporal interference electrical stimulation of the human hippocampus. Nature Neuroscience 26(11): 1994–2004. 37857775 10.1038/s41593-023-01456-8 PMC10620081

[bibr113-23982128251322241] Vlaicu A Bustuchina Vlaicu M (2020) Vagus nerve stimulation for treatment-resistant depression: Is this therapy distinct from other antidepressant treatments? International Journal of Psychiatry in Clinical Practice 24(4): 349–356. 32677482 10.1080/13651501.2020.1779751

[bibr114-23982128251322241] Voigt J Carpenter L Leuchter A (2019) A systematic literature review of the clinical efficacy of repetitive transcranial magnetic stimulation (rTMS) in non-treatment resistant patients with major depressive disorder. BMC Psychiatry 19(1): 13. 30621636 10.1186/s12888-018-1989-z PMC6325728

[bibr115-23982128251322241] Voon V Sun B Wang L , et al (2024) Bed nucleus of the stria terminalis-nucleus accumbens deep brain stimulation for depression: A randomized controlled trial and an intracranial physiological biomarker predictor. Research Square. Available at: https://dx.doi.org/10.21203/rs.3.rs-4854344/v1

[bibr116-23982128251322241] Vöröslakos M Takeuchi Y Brinyiczki K , et al (2018) Direct effects of transcranial electric stimulation on brain circuits in rats and humans. Nature Communications 9: 483. 10.1038/s41467-018-02928-3 PMC5797140 29396478

[bibr117-23982128251322241] Wahab RA Choi M Liu Y , et al (2012) Mechanical bioeffects of pulsed high intensity focused ultrasound on a simple neural model. Medical Physics 39(7): 4274–4283. 22830761 10.1118/1.4729712

[bibr118-23982128251322241] Wessel MJ Beanato E Popa T , et al (2023) Noninvasive theta-burst stimulation of the human striatum enhances striatal activity and motor skill learning. Nature Neuroscience 26(11): 2005–2016. 37857774 10.1038/s41593-023-01457-7 PMC10620076

[bibr119-23982128251322241] White PJ Clement GT Hynynen K (2006) Local frequency dependence in transcranial ultrasound transmission. Physics in Medicine and Biology 51(9): 2293–2305. 16625043 10.1088/0031-9155/51/9/013 PMC1560343

[bibr120-23982128251322241] Widge AS (2024) Closing the loop in psychiatric deep brain stimulation: Physiology, psychometrics, and plasticity. Neuropsychopharmacology 49(1): 138–149. 37415081 10.1038/s41386-023-01643-y PMC10700701

[bibr121-23982128251322241] Wittchen HU Jacobi F Rehm J , et al (2011) The size and burden of mental disorders and other disorders of the brain in Europe 2010. European Neuropsychopharmacology 21(9): 655–679. 21896369 10.1016/j.euroneuro.2011.07.018

[bibr122-23982128251322241] Woodham RD Selvaraj S Lajmi N , et al (2024) Home-based transcranial direct current stimulation treatment for major depressive disorder: A fully remote phase 2 randomized sham-controlled trial. Nature Medicine 31(1): 87–95. 10.1038/s41591-024-03305-y PMC11750699 39433921

[bibr123-23982128251322241] Wu Y Mo J Sui L , et al (2021) Deep brain stimulation in treatment-resistant depression: A systematic review and meta-analysis on efficacy and safety. Frontiers in Neuroscience 15: 655412. 33867929 10.3389/fnins.2021.655412 PMC8047101

[bibr124-23982128251322241] Yaakub SN White TA Roberts J , et al (2023) Transcranial focused ultrasound-mediated neurochemical and functional connectivity changes in deep cortical regions in humans. Nature Communications 14(1): 5318. 10.1038/s41467-023-40998-0 PMC10474159 37658076

[bibr125-23982128251322241] Yan J Pang C Zhou S , et al (2024) 280. Acute temporal interference stimulation of the left dorsolateral prefrontal cortex in patients with depression: First results of a double-blind controlled study. Biological Psychiatry 95(10): S214.

[bibr126-23982128251322241] Yang P-F Phipps MA Jonathan S , et al (2021) Bidirectional and state-dependent modulation of brain activity by transcranial focused ultrasound in non-human primates. Brain Stimulation 14(2): 261–272. 33460838 10.1016/j.brs.2021.01.006 PMC7988301

[bibr127-23982128251322241] Yap JYY Keatch C Lambert E , et al (2020) Critical review of transcutaneous vagus nerve stimulation: Challenges for translation to clinical practice. Frontiers in Neuroscience 14: 284. 32410932 10.3389/fnins.2020.00284 PMC7199464

[bibr128-23982128251322241] Yatsuda K Yu W Gomez-Tames J (2024) Population-level insights into temporal interference for focused deep brain neuromodulation. Frontiers in Human Neuroscience 18: 1308549. 38708141 10.3389/fnhum.2024.1308549 PMC11066208

[bibr129-23982128251322241] Yoo S Mittelstein DR Hurt RC , et al (2022) Focused ultrasound excites cortical neurons via mechanosensitive calcium accumulation and ion channel amplification. Nature Communications 13(1): 493. 10.1038/s41467-022-28040-1 PMC8789820 35078979

[bibr130-23982128251322241] Zadeh AK Raghuram H Shrestha S , et al (2024) The effect of transcranial ultrasound pulse repetition frequency on sustained inhibition in the human primary motor cortex: A double-blind, sham-controlled study. Brain Stimulation 17(2): 476–484. 38621645 10.1016/j.brs.2024.04.005

[bibr131-23982128251322241] Zhai Z Ren L Song Z , et al (2023) The efficacy of low-intensity transcranial ultrasound stimulation on negative symptoms in schizophrenia: A double-blind, randomized sham-controlled study. Brain Stimulation 16(3): 790–792. 37121354 10.1016/j.brs.2023.04.021

[bibr132-23982128251322241] Zhang A Utter D (2018) Remote brain stimulation: A new treatment for Parkinson’s disease? Available at: https://sitn.hms.harvard.edu/flash/2018/remote-brain-stimulation-new-treatment-parkinsons-disease/ (accessed 10 December 2023).

[bibr133-23982128251322241] Zhang Y Zhou Z Zhou J , et al (2022) Temporal interference stimulation targeting right frontoparietal areas enhances working memory in healthy individuals. Frontiers in Human Neuroscience 16: 918470. 36393981 10.3389/fnhum.2022.918470 PMC9650295

[bibr134-23982128251322241] Zhou H Wang M Qi S , et al (2024) Efficacy and safety of transcranial temporal interference stimulation for treating bipolar disorder with depressive episodes. medRxiv. Available at: http://medrxiv.org/content/early/2024/11/20/2024.11.19.24317540.abstract 10.1038/s41380-025-03292-7 41039090

[bibr135-23982128251322241] Zhu Z Yin L (2023) A mini-review: Recent advancements in temporal interference stimulation in modulating brain function and behavior. Frontiers in Human Neuroscience 17: 1266753. 37780965 10.3389/fnhum.2023.1266753 PMC10539552

